# The Novel [4,5-e][1,3]Diazepine-4,8-dione and Acyclic Carbamoyl Imino-Ureido Derivatives of Imidazole: Synthesis, Anti-Viral and Anti-Tumor Activity Evaluations

**DOI:** 10.3390/molecules181113385

**Published:** 2013-10-30

**Authors:** Karlo Wittine, Kristina Poljak, Matea Kovač, Damjan Makuc, Janez Plavec, Jan Balzarini, Tamara Martinović, Sandra Kraljević Pavelić, Krešimir Pavelić, Mladen Mintas

**Affiliations:** 1 Department of Organic Chemistry, Faculty of Chemical Engineering and Technology, University of Zagreb, Marulićev trg 20, Zagreb HR-10000, Croatia; E-Mails: kwittine@fkit.hr (K.W.); polly3112@net.hr (K.P.); matea.kovac@hotmail.com (M.K.); 2 Slovenian NMR Centre, National Institute of Chemistry, Hajdrihova 19, P. O. B. 660, Ljubljana SI-1001, Slovenia; E-Mails: damjan.makuc@ki.si (D.M.); janez.plavec@ki.si (J.P.); 3 EN-FIST Centre of Excellence, Dunajska 156, Ljubljana SI-1001, Slovenia; 4 Rega Institute for Medical Research, KU Leuven, Minderbroedersstraat 10, Leuven B-3000, Belgium; E-Mail: jan.balzarini@rega.kuleuven.be (J.B.); 5 Department of Biotechnology, University of Rijeka, Radmile Matejčić 2, Rijeka 51000, Croatia; E-Mails: tamara.martinovic@uniri.hr (T.M.); sandrakp@biotech.uniri.hr (S.K.); pavelic@biotech.uniri.hr (K.P.)

**Keywords:** imidazole, [4,5-e][1,3]diazepine, anti-RSV, anti-tumor

## Abstract

In the present paper, we report on the synthesis, and *in vitro* antiviral and cytostatic activities of a series of novel imidazole[4,5-e][1,3]diazepine-4,8-dione (compounds **9**–**11**) and acyclic carbamoyl imino-ureido imidazole (compounds **12** and **13**) derivatives. These new type of chemical entities showed no significant activity on the broad spectrum of DNA and RNA viruses. Results of antiproliferative assays performed on a panel of selected human tumor cell lines revealed that only compounds **1** and **5** showed moderate and selective cytostatic effect against HeLa cells (IC_50_ = 24 and 32 µM) with no concomitant cytotoxic effects on human normal fibroblasts (BJ). Importantly, an imidazole derivative containing a pyrrolidine moiety linked via an ethylenic spacer (**3**) showed a selective cytostatic effect toward cervical carcinoma (HeLa) cells (IC_50_ = 9.5 µM) with no apparent cytotoxicity on human normal fibroblasts (BJ). This compound can be therefore considered as a potential anti-tumor lead compound for further synthetic structure optimization.

## 1. Introduction

It has been found that infection with respiratory syncytial virus (RSV), which manifests primarily as bronchiolitis or viral pneumonia, is the leading cause of lower respiratory tract infections (LRTIs) in infants and young children [1]. Ribavirin still is the only antiviral agent approved for the treatment of RSV infection, but due to efficacy and toxicity issues, it has only limited utility [2]. There is a clear need for new anti-RSV therapeutics, with improved efficacy and safety for broader applications [3]. Powell and his colleagues have recently identified a new class of RSV inhibitors, namely 1,4-benzodiazepines [4], which eventually led to identification of RSV604 as a clinical candidate [5].

Furthermore, imidazole is an entity incorporated into many important biological molecules with a wide range of pharmacological activity. In the field of drug discovery the imidazole scaffold is widely used in the drug design strategy and imidazoles are generally well known as anticancer agents as well [6].

Moreover, heterocycles containing an imidazo[4,5-e][1,3]diazepine ring system have already shown potent *in vitro* activity at low micromolar concentrations against lung, breast, ovarian and prostate cancer cell lines [7]. Also, *in vitro* inhibitory activity of a number of ring-expanded nucleosides against NTPase/helicases of a family of *Flaviviridae* have been reported. Compounds containing an imidazo[4,5-e][1,3]diazepine-4,8-dione ring system have exhibited potent activities against HBV and HCV [8]. Ring-expanded nucleosides employing a fused imidazo[4,5-e][1,3]diazepine ring system show promising anti-measles virus activity at submicromolar or micromolar concentration levels with no apparent toxicity to the host cell line [9].

**Figure 1:**
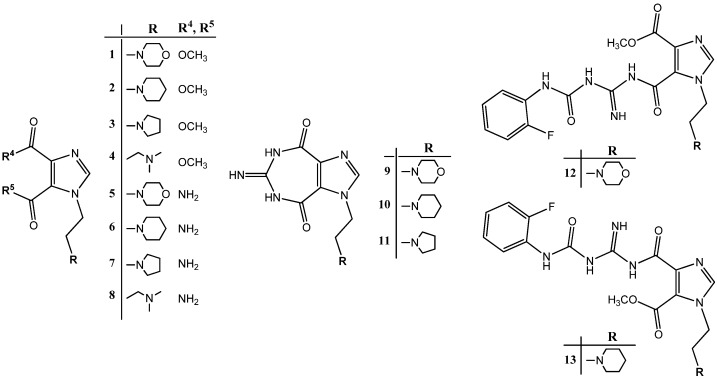
New 1*H*-imidazole-4,5-dicarboxylic acid/amide derivatives (**1**–**8**), imidazo[4,5-e][1,3]diazepine-4,8-dione derivatives (**9**–**11**) and carbamoyl imino-ureido derivatives of imidazole (**12** and **13**).

In the light of these findings we efficiently synthesized a series of new 1*H*-imidazole-4,5-dicarboxylic acid/amide derivatives (**1**–**8**), imidazo[4,5-e][1,3]diazepine-4,8-dione derivatives (**9**–**11**) and carbamoyl imino-ureido derivatives of imidazole (**12** and **13**) (Figure 1) and evaluated their antiviral and cytostatic activity potency.

## 2. Results and Discussion

### 2.1. Chemistry

**Figure 2:**
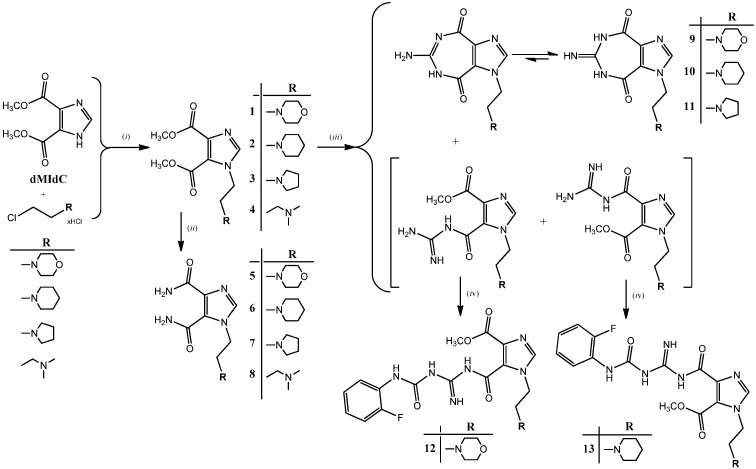
Synthesis of *imidazo[4,5-e][1,3]diazepine-4,8-dione* (**9**–**11**) and *ureido-imino-carbamoyl-imidazole* derivatives (**12**–**13**). Reagents and conditions: (*i*) K_2_CO_3_, CH_3_CN, r.t.; (*ii*) NH_3_, MeOH, r.t.; (*iii*) guanidine hydrochloride, NaOMe, MeOH, r.t.; (*iv*) 2-fluorophenyl isocyanate, DMF, r.t.

The synthesis of new 1*H*-imidazole-4,5-dimethyl dicarboxylate (**1**–**4**), 1*H*-imidazole-4,5-diamide (**5**–**8**), imidazole[4,5-e][1,3]diazepine-4,8-dione (**9**–**11**) and acyclic carbamoyl imino-ureido imidazole derivatives (**12** and **13**) is performed according to the Figure 2.

Reaction of 4,5-dimethyl 1*H*-imidazole-dicarboxylate (**dMIdC**) with K_2_CO_3_ and different 2-chloroalkylamine hydrochlorides, that is 4-(2-chloroethyl)morpholine, 4-(2-chloroethyl)piperidine, 4-(2-chloroethyl)pyrrolidine and 3-dimethylaminopropyl chloride gave 4,5-dimethyl dicarboxylate derivatives (**1**–**4**) with alkylamino chains at the *N*-1 of the imidazole ring. 1*H*-imidazo-4,5-diamide derivatives (**5**–**7**) were obtained by ammonolysis of diesters **1**–**4** in polar protic solvent. Time-controlled reaction of compounds **1**–**3** with guanidine-hydrochloride and sodium-methoxide gave cyclized imidazo[4,5-e]diazepine-4,8-dione derivatives (**9**–**11**) and a mixture of two regioisomeric guanidino carbamoyl-imidazole derivatives which in subsequent *in situ* reaction with 2-fluorophenyl isocyanate in DMF afforded carbamoyl imino-ureido derivatives **12** and **13**. It is worth noting that the reaction of *imidazo[4,5-e][1,3]diazepine-4,8-dione* derivatives (**9**–**11**) with 2-fluorophenyl isocyanate did not gave the desired ureido-imidazo[4,5-e][1,3]diazepine-4,8-dione derivative. The main reason obviously lies in the fact that 6-amino-diazepine-4,8-dione molecules (**9**–**11**) are predominantly in solution in their imino tautomeric form as confirmed by NMR spectroscopy, which is inactive in the final step of the synthesis to create the ureido derivative of imidazo[4,5-e][1,3]diazepine-4,8-dione. Morpholine derivative **12** for which we believed to have a diazepine-4,8-dione structure and whose NMR spectrum indicated next to all diagnostic signals for the assumed structure the presence of an unidentified signal at approximately 3.8 ppm attributable to a OMe group, which is consistent with the results of the determination of the molecular ion mass by high resolution mass spectrometry.

### 2.2. Structural Properties

The structures of **1–13** have been confirmed by ^1^H and ^13^C-NMR spectra (Experimental). The analysis of the spectra was achieved on the basis of the chemical shift, signal multiplicity and integral values. ^19^F NMR resonances of **12** and **13** were well resolved (Experimental). ^1^H decoupled ^13^C-NMR showed C–F coupling constants that enabled straightforward identification of fluorinated carbon atoms and their neighbors. Two sets of signals were observed for **9** in the ratio 4:1 as was estimated from the integral values of ^1^H-NMR signals (e.g., CH-5 proton at *δ* 8.05 and 7.69 ppm). Two species were most probably detected due to presence of dynamic equilibrium between amine and imine tautomers (C8–NH_2_ and C8=NH). C8 chemical shifts of the major species showed value of 149.99 ppm, which is characteristic for the imine form. Therefore, imine form of **9** is the predominant species in solution. Likewise, broad ^13^C-NMR signals suggested presence of tautomer forms for **10** and **11**. Imine forms are favored according to C8 chemical shift (*δ* 149.70 and 150.12 ppm for **10** and **11**, respectively).

The correlation signals observed in ^1^H-^13^C HSQC and HMBC spectra allowed assignment of C2, C3 and C5 atoms. Interestingly, major chemical shifts differences between **12** and **13** were observed for C2 and C3 atoms. The correlation signals between methylene protons CH_2_-1' and C2 and C5 in combination with chemical shift values of imidazole carbon atoms suggested that COOCH_3_ group is attached to C2 (*δ* 121.43 ppm) and fluoro-phenyl-ureido-imino-methyl-carbamoyl moiety to C3 (*δ* 145.53) in **12**. On the other hand, COOCH_3_ in attached to C3 in **13**, which is indicated by C2 and C3 chemical shift (*δ* 127.71 and 134.19 ppm).

These spectroscopic results indicate that compounds **12** and **13** exist as regioisomers with respect to substitution at positions C-2 and C-3 of the imidazole moiety. This implies that both regioisomers are formed as indicated in Figure 2 and their subsequent *in situ* reaction gave compounds **12** and **13**. Structural differences between **12** and **13**, which were suggested by distinct NMR chemical shifts of C2 and C3, were assessed by NOESY experiments. Unfortunately, only trivial NOESY cross-peaks were observed for **12** and **13**. COOCH_3_ group showed no NOESY signals with methylene protons and therefore no particular conformational preferences could be established for these compounds.

### 2.3. Biological Results

#### 2.3.1. Antiviral Activity

Compounds **1**–**3**, **5**–**7** and **9**–**13** were evaluated for their antiviral activity against a wide variety of DNA and RNA viruses, including herpes simplex virus type 1 (HSV-1) (KOS), HSV-2 (G), vaccinia virus and vesicular stomatitis virus in HEL cells, parainfluenza-3, reovirus-1, Sindbis, Coxsackie B4, and Punta Toro virus in Vero cells, vesicular stomatitis virus, Coxsackie virus B4, and respiratory syncytial virus in HeLa cells and influenza A (H1N1; H3N2) and influenza B viruses in MDCK cells. Unfortunately, none of the compounds showed pronounced antiviral activity at subtoxic concentrations. No cytotoxicity for all evaluated compounds on HEL, Vero, HeLa and MDCK cell cultures was observed (data not shown).

#### 2.3.2. Cytostatic Activity

Compounds **1**–**12** were evaluated for their antiproliferative effect against several malignant tumor cell lines: cervical carcinoma (HeLa), colorectal adenocarcinoma, metastatic (SW 620), breast epithelial adenocarcinoma, metastatic (MCF-7) and human hepatocarcinoma (HepG2) cells and compared with their effects on the growth of normal human skin fibroblasts (BJ) (Table 1). The imidazole[4,5-e][1,3]diazepine-4,8-dione derivative linked with a pyrrolidine ligand (**11**) showed a modest cytostatic effect on colon cancer cells (SW620) (IC_50_ = 20 µM) while imidazole derivative containing a pyrrolidine moiety linked via an ethylenic spacer (**3**) showed specific antiproliferative effect on HeLa cells (IC_50_ = 9.5 µM). However, both compounds **11** and **3** exerted moderate growth inhibition only at the highest tested concentrations (10 and 100 µM). Other compounds exerted weak or no antproliferative effects on tested cell lines.

**Table 1:** Inhibitory effects of compounds **1**–**12** on the growth of malignant tumor cell lines in comparison with their effect on normal human skin fibroblasts (BJ).

Tumor cell growth IC_50_^a^ (μM)						
Compd.	Structure	Cell lines				
		MCF-7	HepG2	SW620	HeLa	BJ
**1**	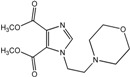	>100	>100	>100	24	>100
**2**	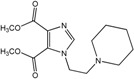	>100	>100	>100	>100	>100
**3**	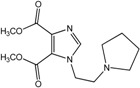	>100	>100	>100	9.5	>100
**4**	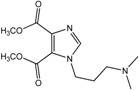	>100	>100	>100	>100	>100
**5**	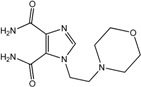	>100	>100	>100	32	>100
**6**	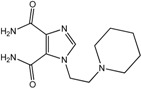	>100	>100	>100	>100	>100
**7**	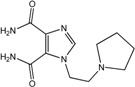	>100	>100	>100	>100	>100
**8**	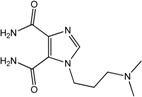	>100	>100	>100	>100	>100
**9**	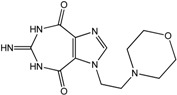	>100	>100	>100	>100	>100
**10**	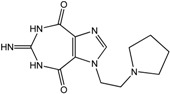	>100	41	31	31	>100
**11**	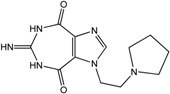	>100	>100	20	>100	>100
**12**	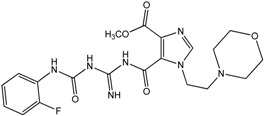	>100	>100	>100	>100	>100

^a^ IC_50_; 50% inhibitory concentration, or compound concentration required to inhibit tumor cell proliferation by 50%.

## 3. Experimental

### 3.1. General Materials and Methods

All commercially available chemicals were purchased from Sigma Aldrich (Hamburg, Germany) and used without purification. All solvents were analytical grade purity and dried. Methanol (CH_3_OH) was stored over 3 Å molecular sieves. Dimethylformamide (DMF) was stored over 4 Å molecular sieves. Dichloromethane (CH_2_Cl_2_) was refluxed over phosphorus pentoxide (P_2_O_5_), distilled and stored over 4 Å molecular sieves. Merck silica gel 60 F_254_ plates were used for thin-layer chromatography. Column chromatography was performed with Merck silica gel (0.063−0.200 mm), with dichloromethane/methanol as eluent. ^1^H and ^13^C-NMR spectra were recorded on a Varian Gemini 300 spectrometer (Institute Ruđer Bošković, Zagreb, Croatia) and Varian NMR System 600 and Varian Unity Inova 300 and Agilent Technologies DD2 300 MHz NMR spectrometers (National Institute of Chemistry, Ljubljana, Slovenia) Samples were measured in CDCl_3_ and DMSO-*d*_6_ solutions at 25 °C in 5 mm NMR tubes. Chemical shifts (*δ*) in ppm were referred to TMS. High performance LC was performed on Agilent 1100 series system with UV detection (photodiode array detector) using Zorbax C18 reverse-phase analytical column (2.1 × 30 mm; 3.5 µm). All compound used for biological evaluation showed >95% purity in this HPLC system. The electron impact mass spectra and the purity of compounds were assessed by using Agilent Technologies 6410 Triple Quad LC/MS instrument equipped with electrospray interface and triple quadrupole analyzer (LC-MS/MS).

### 3.2. Procedures for the Preparations of Compounds

#### 3.2.1. General Procedure for Synthesis of **1**–**4**

To a solution of 4,5-dimethyl 1*H*-imidazole-dicarboxylate (1 g; 5.43 mmol) and K_2_CO_3_ (1.5 g; 10.86 mmol) in CH_3_CN (30 mL) is added 4-(2-chloroethyl)morpholine hydrochloride (for cmpd **1**), 4-(2-chloroethyl)piperidine hydrochloride (for cmpd **2**), 4-(2-chloroethyl)pyrrolidine hydrochloride (for cmpd **3**) or 3-dimethylaminopropyl chloride hydrochloride (for cmpd **4**) (5.43 mmol). Reaction mixture is stirred at room temperature for 48 h. The solvent is evaporated and the crude product simply extracted with CH_2_Cl_2_ or purified by silica gel column chromatography (CH_2_Cl_2_/CH_3_OH = 20/1).

*1-(2-Morpholin-4-yl-ethyl)-1H-imidazole-4,5-dicarboxylic acid dimethyl ester* (**1**): Following general procedure compound **1** is obtained as a yellow oil (1.68 g; 52.23%); MS *m/z*: 297.1 (M+1). ^1^H-NMR (DMSO-*d*_6_): *δ* 2.37 (4H, m, CH_2_-4'), 2.58 (2H, t, *J* = 5.8, CH_2_-2'), 3.52 (4H, m, CH_2_-5'), 3.79 (3H, s, COOCH_3_-3), 3.84 (3H, s, COOCH_3_-2), 4.29 (2H, t, *J* = 5.7, CH_2_-1'), 7.97 ppm (1H, s, CH-5). ^13^C-NMR (DMSO-*d*_6_): *δ* 43.63 (C1'), 52.29 (COOCH_3_-3), 52.86 (COOCH_3_-2), 53.63 (C4'), 58.72 (C2'), 66.57 (C5'), 124.99 (C2), 136.24 (C3), 141.58 (C5), 160.78 (COOCH_3_), 163.44 ppm (COOCH_3_).

*1-(2-Piperidin-1-yl-ethyl)-1H-imidazole-4,5-dicarboxylic acid dimethyl ester* (**2**): Following general procedure compound **2** is obtained as a pale yellow oil (1.32 g; 83.36%); MS *m/z*: 296.2 (M+1). ^1^H-NMR (DMSO-*d*_6_): *δ* 1.25–1.45 (6H, m, 2 × CH_2_-5' and CH_2_-6'), 2.30 (4H, m, CH_2_-4'), 2.51 (2H, t, *J* = 5.7, CH_2_-2'), 3.77 (3H, s, COOCH_3_-3), 3.83 (3H, s, COOCH_3_-2), 4.25 (2H, t, *J* = 5.9, CH_2_-1'), 7.92 ppm (1H, s, CH-5). ^13^C-NMR (DMSO-*d*_6_): *δ* 24.29 (C6'), 25.94 (C5'), 44.09 (C1'), 52.28 (COOCH_3_-3), 52.84 (COOCH_3_-2), 54.47 (C4'), 59.10 (C2'), 125.17 (C2), 136.05 (C3), 141.47 (C5), 160.79 (COOCH_3_), 163.42 ppm (COOCH_3_).

*1-(2-Pyrrolidin-1-yl-ethyl)-1H-imidazole-4,5-dicarboxylic acid dimethyl ester* (**3**): Following general procedure compound **3** is obtained as a pale yellow oil (1.11 g; 72.98%); MS m/z: 282.1 (M+1). ^1^H-NMR (DMSO-*d*_6_): *δ* 1.69 (4H, m, CH_2_-5'), 2.43 (4H, m, CH_2_-4'), 2.70 (2H, t, *J* = 6.1, CH_2_-2'), 3.78 (3H, s, COOCH_3_-3), 3.83 (3H, s, COOCH_3_-2), 4.27 (2H, t, *J* = 6.1, CH_2_-1'), 7.96 ppm (1H, s, CH-5). ^13^C-NMR (DMSO-*d*_6_): *δ* 23.64 (C5'), 45.64 (C1'), 52.30 (COOCH_3_-3), 52.87 (COOCH_3_-2), 53.96 (C4'), 56.20 (C2'), 125.18 (C2), 136.06 (C3), 141.41 (C5), 160.78 (COOCH_3_), 163.39 ppm (COOCH_3_).

*1-(3-Dimethylamino-propyl)-1H-imidazole-4,5-dicarboxylic acid dimethyl ester* (**4**): Following general procedure compound **4** is obtained as a pale yellow oil (0.75 g; 75.0%); MS m/z: 270.1 (M+1). ^1^H-NMR (DMSO-*d*_6_): *δ* 1.84 (2H, m, CH_2_-2'), 2.16 (6H, s, 2 × CH_3_), 2.29 (2H, t, *J* = 6.5, CH_2_-3'), 3.76 (3H, s, COOCH_3_-3), 3.81 (3H, s, COOCH_3_-2), 4.18 (2H, t, *J* = 7.1, CH_2_-1'), 7.96 ppm (1H, s, CH-5). ^13^C-NMR (DMSO-*d*_6_): *δ* 28.21 (C2'), 44.74 (C1'), 45.04 (CH_3_) 52.34 (COOCH_3_-3), 52.99 (COOCH_3_-2), 55.73 (C3'), 124.89 (C2), 136.37 (C3), 141.29 (C5), 160.67 (COOCH_3_), 163.39 ppm (COOCH_3_).

#### 3.2.2. General Procedure for Synthesis of **5**–**8**

To a stirred solution of compounds **1**–**4** (500 mg; 1.68 mmol) in anhydrous CH_3_OH (15 mL) ammonia is introduced at 0 °C. After saturation a reaction mixture is stirred overnight at room temperature. The solvent and excess ammonia are removed under reduced pressure and the crude product purified by silica gel column chromatography (CH_2_Cl_2_/CH_3_OH = 2/1).

*1-(2-Morpholin-4-yl-ethyl)-1H-imidazole-4,5-dicarboxylic acid diamide* (**5**): According to the general procedure compound **5** is obtained as a white crystals (262 mg; 58.17%; m.p. = 196–197 °C); MS *m/z*: 267.1 (M+1). ^1^H-NMR (DMSO-*d*_6_): *δ* 2.39 (4H, m, CH_2_-4'), 2.59 (2H, t, *J* = 6.1, CH_2_-2'), 3.51 (4H, m, CH_2_-5'), 4.53 (2H, t, *J* = 6.1, CH_2_-1'), 7.53 (1H, s, CONH_2_-2), 7.75 (1H, s, CONH_2_-3), 7.88 (1H, s, CH-5), 7.95 (1H, s, CONH_2_-3), 10.65 ppm (1H, s, CONH_2_-2). ^13^C-NMR (DMSO-*d*_6_): *δ* 43.93 (C1'), 53.25 (C4'), 58.51 (C2'), 66.18 (C5'), 126.16 (C2), 136.90 (C3), 140.53 (C5), 160.66 (CO-2), 165.80 ppm (CO-3).

*1-(2-Piperidin-1-il-etil)-1H-imidazol-4,5-diamide* (**6**): According to the general procedure compound **6** is obtained as a white crystals (194 mg; 53.93%; m.p. = 200–201 °C); MS *m/z*: 265.2 (M+1). ^1^H-NMR (DMSO-*d*_6_): *δ* 1.35 (2H, m, CH_2_-6'), 1.43 (4H, m, CH_2_-5'), 2.34 (4H, m, CH_2_-4'), 2.54 (2H, t, *J* = 6.2, CH_2_-2'), 4.51 (2H, t, *J* = 6.2, CH_2_-1'), 7.51 (1H, s, CONH_2_-2), 7.74 (1H, s, CONH_2_-3), 7.85 (1H, s, CH-5), 7.94 (1H, s, CONH_2_-3), 10.64 ppm (1H, s, CONH_2_-2). ^13^C-NMR (DMSO-*d*_6_): *δ* 24.41 (C6'), 26.08 (C5'), 44.90 (C1'), 54.55 (C4'), 59.30 (C2'), 126.64 (C2), 135.36 (C3), 140.98 (C5), 161.14 (CO-2), 166.31 ppm (CO-3).

*1-(2-Piperidin-1-yl-ethyl)-1H-imidazole-4,5-dicarboxylic acid diamide* (**7**): According to the general procedure compound **7** is obtained as a white crystals (182 mg; 45.50%; m.p. = 205–210 °C); MS *m/z*: 252.2 (M+1). ^1^H-NMR (DMSO-*d*_6_): *δ* 1.65 (4H, m, CH_2_-5'), 2.45 (4H, m, CH_2_-4'), 2.72 (2H, t, *J* = 5.6, CH_2_-2'), 4.52 (2H, t, *J* = 6.1, CH_2_-1'), 7.53 (1H, s, CONH_2_-2), 7.75 (1H, s, CONH_2_-3), 7.88 (1H, s, CH-5), 7.94 (1H, s, CONH_2_-3), 10.65 ppm (1H, s, CONH_2_-2). ^13^C-NMR (DMSO-*d*_6_): *δ* 23.16 (C5'), 46.03 (C1'), 53.52 (C4'), 55.97 (C2'), 126.11 (C2), 134.98 (C3), 140.42 (C5), 160.61 (CO-2), 165.80 ppm (CO-3).

*1-(3-Dimethylamino-propyl)-1H-imidazole-4,5-dicarboxylic acid diamide* (**8**): According to the general procedure compound **8** is obtained as a white crystals (144 mg; 48.0%; m.p. = 205–210 °C); MS *m/z*: 240.1 (M+1). ^1^H-NMR (DMSO-*d*_6_): *δ* 1.83 (2H, m, CH_2_-2'), 2.11 (6H, s, 2 × CH_3_), 2.14 (2H, t, *J* = 6.9, CH_2_-3'), 4.42 (2H, t, *J* = 7.0, CH_2_-1'), 7.57 (1H, s, CONH_2_-2), 7.83 (1H, s, CONH_2_-3), 7.89 (1H, s, CH-5), 7.98 (1H, s, CONH_2_-3), 10.66 ppm (1H, s, CONH_2_-2). ^13^C-NMR (DMSO-*d*_6_): *δ* 29.09 (C2'), 45.50 (CH_3_), 45.93 (C3'), 56.31 (C1'), 126.71 (C2), 135.60 (C3), 140.61 (C5), 161.02 (CO-2), 166.25 ppm (CO-3).

#### 3.2.3. General Procedure for Synthesis of **9**–**11**

To a solution of guanidine hydrochloride (5.38 mmol) in anhydrous methanol (10 mL) cooled to 0 °C is added a solution of sodium methoxide (25 wt%; 13 mmol). The reaction mixture is stirred for 30 min at 0 °C. The sodium salt formed is removed by filtration, and the filtrate thus obtained is added to a solution of compounds **1**–**3** (1.35 mmol) in anhydrous methanol (5 mL). Reaction mixture is stirred for 72 h at room temperature. The solvent is evaporated and the crude product purified by silica gel column chromatography (CH_2_Cl_2_/CH_3_OH = 2/1).

*6-Imino-1-(2-morpholin-4-yl-ethyl)-6,7-dihydro-1H,5H-1,3,5,7-tetraaza-azulene-4,8-dione* (**9**): (55 mg; 13.99%; m.p. > 300 °C); MS *m/z*: 292.1 (M+1). ^1^H-NMR (DMSO-*d*_6_): *δ* 2.38 (4H, m, CH_2_-4'), 2.58 (2H, m, CH_2_-2'), 3.51 (4H, m, CH_2_-5'), 4.48 (2H, m, CH_2_-1'), 6.64 (1H, b, NH), 7.37 (1H, b, NH), 8.04 (1H, s, CH-5), 10.66 ppm (1H, b, NH). ^13^C-NMR (DMSO-d_6_): *δ* 43.18 (C1'), 53.19 (C4'), 58.27 (C2'), 66.15 (C5'), 129.7 (C2), 135.2 (C3), 143.41 (C5), 149.99 (C8), 160.27 and 161.63 ppm (C6 and C10).

*6-Imino-1-(2-piperidin-1-yl-ethyl)-6,7-dihydro-1H,5H-1,3,5,7-tetraaza-azulene-4,8-dione* (**10**): (22 mg; 2.23%; m.p. > 300 °C); MS *m/z*: 290.2 (M+1). ^1^H-NMR (DMSO-*d*_6_): *δ* 1.36 (2H, m, CH_2_-6'), 1.43 (4H, m, CH_2_-5'), 2.35 (4H, m, CH_2_-4'), 2.55 (2H, t, *J* = 6.2, CH_2_-2'), 4.46 (2H, t, *J* = 6.2, CH_2_-1'), 6.48 (1H, b, NH), 7.55 (1H, b, NH), 8.02 (1H, s, CH-5), 10.60 ppm (1H, b, NH). ^13^C-NMR (DMSO-d_6_): *δ* 23.86 (C6'), 25.51 (C5'), 43.62 (C1'), 53.98 (C4'), 58.50 (C2'), 130.8 (C2), 133.8 (C3), 143.35 (C5), 149.70 (C8), 161.92 and 162.16 ppm (C6 and C10).

*6-Imino-1-(2-pyrrolidin-1-yl-ethyl)-6,7-dihydro-1H,5H-1,3,5,7-tetraaza-azulene-4,8-dione* (**11**): (55 mg; 13.99%; m.p. > 300 °C); MS *m/z*: 292.1 (M+1). ^1^H-NMR (DMSO-*d*_6_): *δ* 1.90 (4H, m, CH_2_-5'), 3.24 (4H, m, CH_2_-4'), 3.54 (2H, m, CH_2_-2'), 4.68 (2H, t, *J* = 6.1, CH_2_-1'), 6.57 (1H, b, NH), 7.68 (1H, b, NH), 8.19 (1H, s, CH-5), 10.74 ppm (1H, b, NH). ^13^C-NMR (DMSO-d_6_): *δ* 22.65 (C5'), 43.06 (C1'), 53.79 (C4'), 54.14 (C2'), 130.7 (C2), 134.4 (C3), 143.5 (C5), 150.12 (C8), 159.15 and 161.97 ppm (C6 and C10).

#### 3.2.4. General Procedure for Synthesis of **12**–**13**

To a solution of guanidine hydrochloride (514 mg; 5.38 mmol) in anhydrous methanol (8 mL) cooled to 0 °C is added a solution of sodium methoxide (25 wt%; 0.75 mL; 13.12 mmol). The reaction mixture is stirred for 30 min at 0 °C. The sodium salt formed is removed by filtration, and the filtrate thus obtained is added to a solution of compound **1** or **2** (400 mg; 1.35 mmol) in anhydrous methanol (5 mL). Reaction mixture is stirred for 8 h at room temperature, solvent evaporated and in reaction mixture *in situ* dissolved in DMF (3 mL) is added 2-fluorophenyl isocyanate (0,02 mL; 0,18 mmol). Reaction mixture is stirred for 24 h at room temperature. The solvent is evaporated and the crude product purified by silica gel column chromatography (CH_2_Cl_2_/CH_3_OH = 60/1) to give:

*5-({[3-(2-Fluoro-phenyl)-ureido]-imino-methyl}-carbamoyl)-1-(2-morpholin-4-yl-ethyl)-1H-imidazole-4-carboxylic acid methyl ester* (**12**) as a white crystals (6.5 mg; 1.63%; m.p. > 164–165 °C); MS *m/z* 462.1915 (M+1). ^1^H-NMR (DMSO-*d*_6_): *δ* 2.48 (4H, t, *J* = 4.7, CH_2_-4'), 2.73 (2H, t, *J* = 6.0, CH_2_-2'), 3.66 (4H, t, *J* = 4.7, CH_2_-5'), 4.10 (3H, s, COOCH_3_-3), 4.55 (2H, t, *J* = 6.0, CH_2_-1'), 6.97 (1H, m, F-C_6_H_4_), 7.06 (1H, m, F-C_6_H_4_), 7.11 (1H, m, F-C_6_H_4_), 7.37 (1H, b, NH), 7.71 (1H, s, CH-5), 8.29 (1H, m, F-C_6_H_4_), 8.83 (1H, b, NH), 9.57 (1H, b, NH), 12.48 ppm (1H, b, NH). ^13^C-NMR (DMSO-*d*_6_): *δ* 24.11 (C6'), 25.71 (C5'), 43.49 (C1'), 54.29 (C4'), 59.05 (C2'), 115.10 (F-C_6_H_4_, d, *J*_CF_ = 18.0), 116.5 (F-C_6_H_4_), 121.43 (C2), 124.80 (F-C_6_H_4_, d, *J*_CF_ = 3.2), 136.3 (F-C_6_H_4_), 139.97 (C5), 145.53 (C3), 150.95 (F-C_6_H_4_, d, *J*_CF_ = 236.4), 158.20, 161.73 and 163.60 (2×C=O and C=NH), 167.55 (COOCH_3_). ^19^F NMR (DMSO-*d*_6_): *δ* −125.27 (F-C_6_H_4_, b).

*5-({[3-(2-Fluoro-phenyl)-ureido]-imino-methyl}-carbamoyl)-3-(2-piperidin-1-yl-ethyl)-3H-imidazole-4-carboxylic acid methyl ester* (**13**) as a yellow oil (7.2 mg; 0.72%); MS *m/z* 460.2 (M+1). ^1^H-NMR (CDCl_3_): *δ* 1.34 (2H, m, CH_2_-6'), 1.42 (4H, m, CH_2_-5'), 2.30 (4H, m, CH_2_-4'), 2.5 (2H, m, CH_2_-2'), 3.73 (3H, s, COOCH_3_-2), 4.17 (2H, t, *J* = 6.2, CH_2_-1'), 6.5–7.0 (4 × 1H, m, F-C_6_H_4_), 7.69 (1H, s, CH-5), 7.7–8.6, 12.3 ppm (4 × 1H, b, NH). ^13^C-NMR (CDCl_3_): *δ* 46.15 (C1'), 53.79 (C4'), 53.86 (COOCH_3_), 58.60 (C2'), 66.90 (C5'), 114.70 (F-C_6_H_4_, d, *J*_CF_ = 19.2), 120.47 (F-C_6_H_4_), 122.66 (F-C_6_H_4_, d, *J*_CF_ = 7.1), 124.36 (F-C_6_H_4_, d, *J*_CF_ = 3.3), 127.69 (F-C_6_H_4_, d, *J*_CF_ = 16.5), 127.71 (C2), 134.19 (C3), 142.33 (C5), 152.25 (F-C_6_H_4_, d, *J*_CF_ = 242.8), 158.04, 160.43 and 162.98 (2×C=O and C=NH), 165.70 (COOCH_3_). ^19^F NMR (CDCl_3_): *δ* −124.37 (F-C_6_H_4_, b).

### 3.3. Biological Methods

#### 3.3.1. Cell Culturing

The cell lines HeLa (cervical carcinoma), SW620 (colorectal adenocarcinoma, metastatic), MCF-7 (breast epithelial adenocarcinoma, metastatic), HepG2 (hepatocellular carcinoma) and BJ (normal diploid human fibroblasts), were cultured as monolayers and maintained in Dulbecco’s modified Eagle medium (DMEM) supplemented with 10% fetal bovine serum (FBS), 2 mM L-glutamine, 100 U/mL penicillin and 100 μg/mL streptomycin in a humidified atmosphere with 5% CO_2_ at 37 °C.

#### 3.3.2. Proliferation Assays

The panel cell lines were inoculated onto a series of standard 96-well microtiter plates on day 0, at 3,000 to 5,000 cells per well according to the doubling times of specific cell line. Test agents were then added in 10-fold dilutions (0,01 to 100 µM) and incubated for further 72 h. Working dilutions were freshly prepared on the day of testing in the growth medium. The solvent (DMSO) was also tested for eventual inhibitory activity by adjusting its concentration to be the same as in the working concentrations (DMSO concentration never exceeded 0.1%). After 72 h of incubation, the cell growth rate was evaluated by performing the MTT assay: experimentally determined absorbance values were transformed into a cell percentage growth (PG) using the formulas proposed by NIH and described previously [10]. This method directly relies on control of untreated cells at the day of substances addition because it compares the growth of treated cells with the growth of untreated cells in control wells on the same plate—the results are therefore a percentile difference from the calculated expected value. The IC_50_ values for each compound were calculated from dose-response curves using linear regression analysis by fitting the mean test concentrations that give PG values above and below the reference value. If, however, all of the tested concentrations produce PGs exceeding the respective reference level of effect (e.g., PG value of 50) for a given cell line, the highest tested concentration is assigned as the default value (in the screening data report that default value is preceded by a “>” sign). Each test point was performed in quadruplicate in three individual experiments. The results were statistically analyzed (ANOVA, Tukey post-hoc test at *p* < 0.05). Finally, the effects of the tested substances were evaluated by plotting the mean percentage growth for each cell type in comparison to control on dose response graphs.

#### 3.3.3. Antiviral Activity Assays

The antiviral assays, other than the anti-HIV assays, were based on inhibition of virus-induced cytopathicity in HEL [herpes simplex virus type 1 (HSV-1) (KOS), HSV-2 (G), vaccinia virus and vesicular stomatitis virus], Vero (parainfluenza-3, reovirus-1, Sindbis, Coxsackie B4, and Punta Toro virus), HeLa (vesicular stomatitis virus, Coxsackie virus B4, and respiratory syncytial virus) or MDCK [influenza A (H1N1; H3N2) and influenza B] cell cultures. Confluent cell cultures (or nearly confluent for MDCK cells) in microtiter 96-well plates were inoculated with 100 CCID_50_ of virus (1 CCID_50_ being the virus dose to infect 50% of the cell cultures). The cell cultures were incubated at the time of infection in the presence of varying concentrations (200, 40, 8, … µM) of the test compounds. Viral cytopathicity was recorded microscopically as soon as it reached completion in the control virus-infected cell cultures that were not treated with the test compounds. The methodology of the anti-HIV assays was as follows: human CEM cells (~3 × 10^5^ cells/mL) were infected with 100 CCID_50_ of HIV(III_B_) or HIV-2(ROD)/mL and seeded in 200-µL wells of a microtiter plate containing appropriate dilutions of the test compounds. After 4 days of incubation at 37 °C, HIV-induced giant cell formation was examined microscopically.

## 4. Conclusions

The main objective of this study was synthesis of a molecule that would be closely related to RSV604. None of the compounds showed pronounced antiviral activity at subtoxic concentrations. No cytotoxicity for all evaluated compounds on HEL, Vero, HeLa and MDCK cell cultures was observed.

From the structure-activity point of view, we believe that the main reason for lack of antiviral activity is probably the absence of either sugar moiety at *N*-1 of imidazole or a closer resemblance to the active compound RSV604, that was chemically caused and constrained by the formation of the inactive imino form of the cyclized product instead of the targeted amino form. The 1*H*-imidazole-4,5-dicarboxylic acid dimethyl ester derivative having an ethyl morpholino ligand (compound **1**) and the 1*H*-imidazole-4,5-diamide derivative with morpholino moiety bound to an imidazole ring (compound **5**) have also exerted selective but modest cytostatic effect towards human cervix carcinoma (HeLa) cells (IC_50_ = 24 and 32 µM), while moderate non-selective antiproliferative activity was observed for compound **10**. The imidazole[4,5-e][1,3]diazepine-4,8-dione derivative linked with a pyrrolidine ligand (**11**) showed only a modest cytostatic effect on colon cancer cells (SW620) (IC_50_ = 20 µM). These compounds showed no cytotoxic effects on human normal fibroblasts.

Synthetic chemistry is often full of surprises and presented compounds containing a carbamoyl imino-ureido moiety are structurally very interesting, e.g., compounds **12** and **13**. Moreover, 1*H*-imidazole derivative containing a pyrrolidine moiety linked via an ethylenic spacer to *N*-1 of the imidazole ring (compound **3**) exerted a more pronounced selective cytostatic effect towards human cervix cancer (HeLa) cells (IC_50_ = 9.5 µM) in comparison with other tested compounds with no apparent cytotoxicity on normal human skin fibroblasts (BJ) as well. This compound might therefore be suited for further exploration as a potential lead compound and chemical modifications.
